# Correlation between breastfeeding and maternal health status

**DOI:** 10.1590/S1679-45082013000200008

**Published:** 2013

**Authors:** Carlos Zubaran, Katia Foresti

**Affiliations:** 1 University of Western Sydney, Sydney, Australia; 2 Universidade de Caxias do Sul, Caxias do Sul, RS, Brazil

**Keywords:** Breastfeeding, Self-efficacy, Health status, Questionnaires, Brazil

## Abstract

**Objective:**

To examine the relation between breastfeeding efficacy and health status in a sample of 88 mothers from Southern Brazil.

**Methods:**

Research participants completed the Portuguese version of the General Health Questionnaire and the Personal Health Scale. Breastfeeding efficacy was evaluated using the Breastfeeding Self-Efficacy Scale. Correlations between breastfeeding and health status scores were examined using Pearson's correlation coefficient.

**Results:**

The results of this study revealed significant correlations between the scores of the two general health and well-being questionnaires and the Breastfeeding Self-Efficacy Scale. Bivariate regression analyses revealed that both health status instruments significantly predicted Breastfeeding Self-Efficacy Scale scores.

**Conclusion:**

Breastfeeding efficacy is significantly related to maternal health status in Southern Brazil.

## RESUMO

**Objetivo:**

Examinar a relação entre eficácia do aleitamento e o estado de saúde em uma amostra de 88 mães na Região Sul do Brasil.

**Métodos:**

As participantes da pesquisa completaram a versão em português do Questionário de Saúde Geral e da Escala de Saúde Pessoal. A eficácia do aleitamento foi avaliada por meio da Escala de Autoeficácia no Aleitamento. As correlações entre o escore do questionário referente ao aleitamento e os instrumentos de avaliação do estado de saúde foram examinadas usando-se o coeficiente de correlação de Pearson.

**Resultados:**

Houve correlação significante entre os escores dos questionários de estado de saúde e da escala de eficácia do aleitamento. Análises de regressão bivariada revelaram valores preditivos significantes de ambos os instrumentos de avaliação do estado de saúde sobre os escores da escala de eficácia do aleitamento.

**Conclusão:**

A eficácia do aleitamento está relacionada ao estado de saúde materno no sul do Brasil.

## INTRODUCTION

Breastfeeding is an important component of the maternal experience^(1)^. The scientific basis of the benefits of breastfeeding is well-established and recent policies and guidelines encourage health professionals to promote it^(2)^. In 2001 a World Health Organization (WHO) directive recommended breastfeeding to be continued for a period of at least to 2 years, with the introduction of weaning foods after an initial 6-month period of exclusive breastfeeding^(3)^.

Breastfeeding is associated with developmental and health benefits for the infant^(4)^ and favors the bonding experience between the mother and the baby^(5)^. Also, breastfeeding and lactation produce important maternal benefits^(6)^, such as decreased postpartum bleeding^(7)^, reduced incidence of type 2 diabetes^(8)^ and reduced risk of breast^(9)^ and ovarian cancer^(10)^.

In addition to physiological factors, the breastfeeding experience is influenced by sociocultural and economic features^(11)^. Mothers experience significant health changes during the postpartum and breastfeeding periods, including fatigue, headache, dyspareunia, haemorrhoids and pain at multiple sites^(12)^. A percentage of mothers also experience psychological distress and dysphoria^(1,13)^.

These dysfunctions are often regarded as transient and inconsequential. Yet, these changes are associated with significant functional impairment and poor maternal emotional status^(14)^. Some mothers may experience the recurrent demands of a breastfeeding child as physically and emotionally exhausting^(13)^, which may bring about sensations of loss of control and low self-esteem^(15)^. Such challenges may cause guilty feelings and doubts about the continuation of breastfeeding^(16)^.

Although human lactation involves physical and psychological aspects, there is limited scientific evidence about the interplay between maternal health status and breastfeeding efficacy.

To the authors' knowledge, this is the first study to explore the correlations between maternal health status and breastfeeding efficacy in Brazil.

## OBJECTIVE

The aim of the present study was to examine this relation in a sample of mothers from Southern Brazil. In this study, the scores of two health status questionnaires were correlated with the scores of a breastfeeding efficacy scale.

## METHODS

### Sample

All participants were recruited from the *Universidade de Caxias do Sul* general hospital in Southern Brazil. It is a teaching hospital and a regional referral center in obstetrics. The hospital is certified by the WHO Baby-Friendly Hospital Initiative^(17)^.

Eligible participants were all hospitalized breastfeeding mothers who were able to understand Portuguese and had given birth to a healthy infant. Research volunteers were invited via word of mouth by members of the research team, in the days following delivery, while still at hospital. A complete description of the research protocol was given to prospective participants. Mothers were excluded if they presented with factors that could significantly interfere with breastfeeding, such as multiple births or high-risk pregnancy (i.e., serious medical conditions or known birth defect), or if the baby had not been discharged from the hospital with the mother. Following the initial screening, 101 women were considered eligible and agreed to participate in the study. During the home visit interview 12 women were no longer breastfeeding due to several reasons. One participant did not complete the Personal Health Scale (PHS) questionnaire and was excluded from the sample. The final sample included all mothers who were still breastfeeding and completed all questionnaires (n=88) representing 88.9% of the overall potentially eligible sample.

### Recruitment procedures

Potential participants were identified by the research team 1 to 3 days after delivery, and approached for further eligibility assessment and explanation about the study. After informed consent procedures, a demographic questionnaire was completed before hospital discharge. Given that a substantial fraction of the sample was illiterate or semiliterate, questionnaires were verbally administered to some women. In these cases, minor clarifications were eventually made by trained examiners who followed standardized instructional procedures. All mothers were interviewed in their homes once, between the 2^nd^ and 12^th^ week postpartum, and completed the Portuguese version of the questionnaires.

### Informed consent

This study was approved by the Institutional Ethics and Research Committee of the *Universidade de Caxias do Sul*. All participants signed a consent form declaring their voluntary agreement to all procedures involved in the project.

### Instruments

#### The Breastfeeding Self-Efficacy Scale – Short Form

The Breastfeeding Self-Efficacy Scale – Short Form (BSES-SF) is a 14-item self-report instrument developed to assess breastfeeding confidence^(18)^. The BSES-SF is an ordinal scale in which all items are preceded by the phrase “I can always” and assessed on a 5-point Likert-type scale in which the number 1 corresponds to “not at all confident” and the number 5 to “always confident”. All items are positively keyed and higher scores indicate higher levels of breastfeeding self-efficacy. The BSES-SF was validated in several languages, including Portuguese^(19)^.

#### General Health Questionnaire

The General Health Questionnaire (GHQ) is a widely used instrument that has been extensively employed in various settings since its development by Goldberg, in the 1970's^(20)^. The questionnaire was originally developed as a 60-item instrument, although shortened versions including GHQ-30, GHQ-28, GHQ-20, and GHQ-12 are currently available. The scale asks whether the respondent has experienced a particular symptom or behavior over the preceding few weeks. Each item is rated on a 4-point scale (less than usual, no more than usual, rather more than usual, or much more than usual). GHQ-12 is brief, simple and easy to complete and its application as a screening tool in research in a wide variety of clinical groups, of different cultures, has been well documented^(21,22)^. The scale has been shown to have good reliability and validity^(20)^. It has also been used to screen for postnatal depression^(23)^ and is used routinely in obstetric practice and research in Hong Kong^(23,24)^. High overall GHQ scores suggest low health status.

#### Personal Health Scale

The Personal Health Scale (PHS) is a concise instrument for comprehensive culture-informed and self-rated assessment of general health status and well-being. It is composed of ten questions that appraise different health status dimensions, including aspects such as somatic and psychological status and social functioning. In the development of the PHS, special attention was given to cultural diversity^(25)^ and different versions were validated in several languages^(25)^. The higher the overall PHS score, the lower the health status.

The Portuguese version of the PHS^(25)^ was used in this study. In the validation study, the original English version was successfully adapted to Portuguese and the authors concluded that it constitutes a reliable and trustworthy research instrument for evaluation of health status in Brazil.

#### Socioeconomic status measurement scale

All research participants completed a socioeconomic status (SES) scale that had been previously developed and tested in Brazil^(26)^. Based on this instrument, participants were allocated to one of six socioeconomic strata: lower-lower class, upper-lower class, lower-middle class, middle class, upper-middle class and upper class.

### Statistical analysis

Demographic analysis was performed to examine age, schooling level, SES and number of previous pregnancies and deliveries of research participants. Metrics from different questionnaires were converted to Z-scores for comparative analyses. Relevant correlations between different assessment tools were examined using Pearson product-moment correlation coefficients. Score means of various groups according to different variables were compared using the *t* test. Finally, bivariate linear regression analyses were performed to check the predictive value of health status questionnaires (PHS and GHQ) scores over breastfeeding efficacy scores (BSES-SF). All statistical analyses were performed using Statistical Package for the Social Science^®^ (SPSS^®^).

### Ethics approval

This study was approved by the Ethics and Research Committee of the *Universidade de Caxias do Sul*, protocol number 64.

## RESULTS

### Demographic statistics

The age of the participants ranged from 14 to 42 years (mean age of 25.4 years; SD=6.99). Women were classified according to Brazilian census criteria as white (n=67; 76.1%), black (n=8; 9%) or brown (n=13; 14.8%). Marital status data revealed that 37.5% (n=33) of the mothers were in *de facto* relationships (common-law marriages), 37.5% (n=33) were married, 22.7% (n=20) were single and 2.3% (n=2) were divorced. Most mothers (n=58; 66%) had delivered vaginally and 34% (n=30) had undergone cesarean delivery. On average, research participants were interviewed 7.69 (±1.7 SD) weeks after delivery.

Most mothers (43.2%, n=38) in the study had given birth to their first child. Of the remaining mothers, 29.5% (n=26) had given birth to their second child, 14.8% (n=13) to their third, and 12.5% (n=11) to their fourth or subsequent child respectively. At the time of interview, 68 participants (77.3%) were exclusively breastfeeding and 20 (22.7%) were partially breastfeeding. SES distribution in the sample studied was as follows: 1% (n=1) of lower-lower class, 15% (n=13) of upper-lower class, 72% (n=63) of lower-middle class and 12% (n=11) of middle class women. None of the participants were of upper-middle or upper class. One participant (1%) held a university degree; 4 (4.5%) reported incomplete higher education; 18 (20%) had a high school diploma; 22 (25%) had not finished high school; 5 (6%) had completed primary school; and 38 (43%) had incomplete basic education.

### Breastfeeding efficacy

The mean BSES-SF score of the entire sample was 63.3 (SD=6.3; range=43-70). The mean score (SD) of mothers who exclusively breastfed (n=68) was 65.6 (SD=4), whereas the score of mothers who combined breastfeeding and formula (n=20) was 55.8 (SD=7). The mean scores of primiparous (n=38) and multiparous women were 63.6 (SD=6.6) and 63.3 (SD=6.2), respectively.

### Breastfeeding efficacy and other obstetric variables

Analysis of variance (ANOVA) was conducted to compare BSES-SF score means according to different variables. The mean overall BSES-SF score of women who exclusively breastfed was significantly higher than those of mothers who combined breastfeeding with formula [t(2,86)=5.9; p<0.001]. No significant BSES-SF score differences were observed between primiparous and multiparous women (p=0.85). The presence or absence of clinical complications during pregnancy had no significant impact (p=0.29) on BSES-SF scores and no significant differences were observed between the mean BSES-SF scores according to type of delivery (vaginal *versus* cesarean section). Mean BSES-SF scores did not differ significantly according to health status (healthy *versus* unhealthy) of the newborn (p=0.84), alcohol consumption and smoking during pregnancy (p=0.5) or history of depression (p=0.5). However, a significant difference was observed between mothers who had an intimate partner and those who were not involved in a relationship [t(2, 86)=- 2,22; p=0.03].

### Health status

Mean PHS and GHQ scores according to type of breastfeeding and parity are displayed in table 1. No significant differences were observed between the mean PHS and GHQ scores of mothers who exclusively breastfed and mothers who combined breastfeeding with formula (p=0.25, respectively). Mean PHS scores of primiparous and multiparous mothers did not differ significantly (p=0.07). No significant differences were documented between mean PHS (p=0.49) and GHQ (p=0.43) scores according to the health status (healthy *versus* unhealthy) of the newborn, alcohol consumption and smoking during pregnancy (p=0.97 for PHS; p=0.88 for GHQ) or history of depression (p=0.52 for PHS; p=0.11 for GHQ). Conversely, mean GHQ scores differed significantly between the two groups of mothers, according to parity (p=0.02).

**Table 1:** Personal Health Scale (PHS) and General Health Scale (GHQ) scores according to type of breastfeeding and parity

Assessment tool			Exclusive breastfeeding	Combined breastfeeding	Primiparous	Multiparous	Total sample
		n	68	20	38	50	88
PHS							
	Mean (SD)		5.6 (4.5)	6.9 (4.7)	4.9(4)	6.6 (4.8)	5.9 (4.5)
	Range (min-max)		18 (0-18)	16 (0-16)	15 (0-15)	18 (0-18)	18 (0-18)
GHQ							
	Mean (SD)		23.8 (6.5)	25.8 (7.7)	22.3 (5.5)	25.7 (7.4)*	24.3 (6.8)
	Range (min-max)		31 (15-46)	31 (14-45)	20 (14-34)	32 (14-46)	22 (14-46)

* Statistical significance (p=0.02).

SD = standard deviation.

### Breastfeeding efficacy and health status

Significant correlations (r) were observed between the overall BSES-SF scores and the scores of PHS and GHQ. As expected, the correlation between both health status questionnaires was significantly high (r=0.76; p<0.001). Significant correlations (p<0.001) between the BSES-SF and the PHS and GHQ scores were 0.37 and 0.38 respectively.

**Figure 1:**
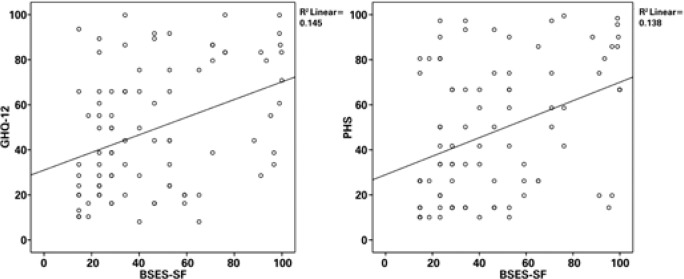
Scatter plots of the bivariate linear regressions of the Z-scores of the Breastfeeding Self-Efficacy Scale overall ratings based on the General Health Questionnaire (GHQ-12) and the Personal Health Scale (PHS)

Bivariate linear regression analyses were conducted to assess the predictive value of PHS and GHQ scores over breastfeeding efficacy (BSES-SF) scores. PHS and GHQ scores significantly predicted BSES-SF scores [R^2^=0.14, F (1,86)=13.77; p<0.001 and R^2^=0.14, F (1,86)=14.56; p<0.001 respectively] (Figure 1).

## DISCUSSION

According to demographic results most women evaluated in this study were white, in their mid-twenties, had limited educational background and came from the lower SES echelons of the Brazilian society. Most mothers were living in a *de facto* relationship and breastfeeding her first baby following a vaginal delivery. The sample represented the typical women seeking obstetric care in public health services in South Brazil, as lower echelons of the Brazilian society constitute the usual population attending public hospitals in the country^(27)^.

The mean BSES-SF score of the Brazilian sample (63.3) was higher than the mean scores of Polish (55.6) and Turkish (60.1) samples but lower than the scores of Chinese (118.8) and Spanish (131.8) samples. The mean score documented in the Brazilian sample is therefore within reasonable distribution parameters, suggesting that Brazilian women in this study had intermediate levels of breastfeeding efficacy. Based on the results of this study maternal and neonatal characteristics are not significantly responsible for different levels of breastfeeding efficacy, given neither parturition nor history of obstetric and neonatal complications significantly influenced BSES-SF scores.

The mean PHS score in this sample (5.9) was slightly below the cutoff score established for this questionnaire in Brazil (6)^(25)^. Therefore, based on the PHS, maternal health status in this sample was marginally lower than the expected score for individuals in good health. The mean GHQ score observed in this sample (±11) was similar to the score of a sample of Belgian women who completed the same questionnaire between 1 and 4 months following delivery (±12)^(28)^. The mean scores observed in this study were significantly higher than the screening GHQ-12 score proposed for morbidity (4/5)^(29)^.

GHQ was considered a valuable screening tool for detection of postpartum depression, anxiety and adjustment disorders when conjointly tested. The GHQ-12 has been recommended as an easily administered and accurate screening tool for the identification of individuals with potential non-psychotic psychiatric disturbances.

Based on the results of this study, breastfeeding self-efficacy is correlated with health status, as demonstrated by the significant correlations between the BSES-SF and the two questionnaires that measure health status and well-being (PHS and GHQ). The associations observed are strong enough to predict the relation between health status and breastfeeding self-efficacy.

A positive association between quality of life and duration of breastfeeding has been previously reported^(8)^. In this study, mothers who breastfed for ±6 months reported significantly higher health-related quality of life scores than those who did not breastfeed, including physical functioning (p=0.046), general health perception (p<0.001) and mental health (p=0.026).

Finally, this study supports previous results indicating that mothers who exclusively breastfeed have significantly higher well-being status than mothers who combine breastfeeding with formula^(8)^. Given the limited information available in this field, further studies investigating the interface between health status and maternal behavior during the postpartum period are required^(30)^. The scarcity of instruments designed to investigate maternal physical and mental health in the postpartum period prevents systematic comparative analyses at the national international levels^(30)^. This study endeavors to contribute to the advancement of knowledge in a needy research area, particularly where vulnerable populations (women of low SES living in developing economies, such as Brazil) are concerned.

This study may present limitations, including potential biases related to sampling procedures and study design. In this study, research volunteers were recruited exclusively from a public hospital. In Brazil significant inequalities between the private and the public health care sectors epitomize abysmal socioeconomic differences, in that professionals and their respective family members seek predominantly private hospitals, while workers of lower income are overrepresented in public hospitals^(27)^. It is therefore plausible to infer that, due to discrete levels of financial deprivation, the health status of the sample studied may be lower than that of the more affluent segments of the Brazilian population. The findings in this study may therefore not be fully applicable to the entirety of the Southern Brazilian population. Also, the cross-sectional design of this study precludes causal interpretations of the results presented.

Given the convenience sampling method used in this study, generalizations to a wider population may not necessarily have scientific value. However, given that the public health care facility where this study was conducted is of similar standard to other public obstetric health care centers in the region, this particular sample should be expected to behave as a random sample of the same population.

## CONCLUSION

The results of this study show that breastfeeding efficacy is significantly related to health status and well-being among mothers in South Brazil. The association between health status and breastfeeding efficacy, as measured by two questionnaires employed in this study, was significant and had predictive value. The results described indicate that the assessment of maternal health status may be helpful to gauge the breastfeeding efficacy. Moreover, they could warn healthcare professionals to mothers predicted to have difficult breastfeeding experiences. These results support previous evidences demonstrated by the same research group that health status is an important element to be factored in the diagnosis of postnatal depression. Additional studies are required to further test the value of health status assessment tools in predicting breastfeeding efficacy in larger samples. Finally, the results of the present study indicate that health status measures and breastfeeding efficacy are valid research topics in community health care programs.
